# Dietary Fiber Intake among Normal-Weight and Overweight Female Health Care Workers: An Exploratory Nested Case-Control Study within FINALE-Health

**DOI:** 10.1155/2017/1096015

**Published:** 2017-11-13

**Authors:** Jenny Hadrévi, Karen Søgaard, Jeanette Reffstrup Christensen

**Affiliations:** ^1^Department of Community Medicine and Rehabilitation, Umeå University, 901 87 Umeå, Sweden; ^2^Department of Sports Science and Clinical Biomechanics, University of Southern Denmark, Campusvej 55, 5230 Odense, Denmark; ^3^Department of Clinical Research, University of Southern Denmark, Kløvervænget 3, Indgang, 5000 Odense, Denmark; ^4^Department of Public Health, University of Southern Denmark, JB Winsløwsvej 9A, 5000 Odense, Denmark

## Abstract

Socioeconomic factors affect choice of diet, that is, dietary fiber intake. Underreporting of food consumption in diet surveys has been reported higher in low-income, low-education groups compared to high-income, high-education groups. This paper examines in a socioeconomic homogenous low-income low-education group of females the relation between dietary fiber intake and overweight and scrutinizes if the level of underreporting is equally large in normal-weight and overweight groups. Thirty-four female health care workers classified as either normal-weight (*N* = 18) or obese (*N* = 16) based on BMI, fat percentage, and waist circumference participated. A detailed food-diary was used to record their dietary intake in 9 days. Average dietary fiber intake in the normal-weight group was 2.73 +/− 0.65 g/MJ, while it was 2.15 +/− 0.64 g/MJ for the women in the obese group. In both groups, the overall food intake was underreported. In spite of a significantly lower dietary fiber intake in the obese group, the present population of women working within health care all showed an overall low dietary fiber intake and a general underreporting of food intake. These results indicate a clear need for dietary advice especially on fiber intake to increase general health and decrease weight.

## 1. Introduction

The prevalence of obesity is increasing and today more than half of the world's population is considered to be overweight [1]. The increased overweight prevalence within the general population may partly be due to an increased consumption of processed foods with a low intake of dietary fiber [2]. Previous studies have shown a correlation between low dietary fiber intake and overweight [3, 4].

Ingestion of a certain amount of dietary fiber apart from alleviating constipation also reduces hunger, thereby reducing total energy intake and preventing weight gain. In line with this, previous interventions on weight reduction have shown a positive effect of increase in dietary fiber intake on weight loss [5].

During the years there has been plenty of information regarding the positive effects on dietary fiber and general health. Knowledge is now that fiber products protect against colon cancer, breast cancer, diabetes, and cardiovascular diseases (CVD) [6, 7]. This has led governments and health organizations around the world to establish recommendations for daily dietary fiber intake [8].

A high proportion of individuals working in low-income low-education jobs such as health care workers are overweight or obese [9]. Low socioeconomic status and low educational level are correlated with unhealthy eating habits such as low intake of dietary fiber [10].

Earlier interventions among low-income occupations with hard work exposures aiming to increase general health and decrease risk of lifestyle diseases have primarily consisted of physical exercise and ergonomics; but dietary advices may be equally relevant to consider in future interventions [11]. However, studies investigating the relation between overweight and intake of dietary fibers within a group with the same socioeconomic status and the same daily work exposure are scarce.

In general, a certain level of underreporting of food intake is present in diet surveys but in low-income low-education groups, the underreporting has been shown to be particularly high compared to groups with high income and high education [12]. However, few studies have considered underreporting in diet, particularly within a population of low-income and low-education jobs [12, 13] and if their reporting is biased by overweight and obesity [14]. Such information on the reliability of data on eating habits is however crucial for the tailoring of the proper diet and exercise intervention within these job groups.

The aim of the present study is to investigate the relationship between dietary fiber and obesity within a group of low-income low-education female health care workers. The correlation between body composition and dietary fiber intake is evaluated using a nine-day dietary record and risk of bias is considered by an estimation of the level of potential underreporting. Previous studies have stipulated underreporting in low socioeconomic groups; here we scrutinize the level of underreporting in a representative group with the aim of elucidating if underreporting can be generalized within the group.

## 2. Materials and Methods

### 2.1. Subjects

The study consisted of a subsample from a population of 98 participants from the FINALE-Health study [16], a cluster randomized single-blinded controlled trial conducted from May 2009 to the end of June 2010. The subsample entails 34 Danish female health care workers (aged 28 to 60) all employed in the same local care center. The ethnicity of the population is Caucasian and the Danish society is an industrialized and rich society without poverty; hence starvation is not adequate in low-income groups but rather overweight and related health problems. The inclusion criteria for the subgroup were defined based on their body composition, that is, Body Mass Index (BMI), body fat percentage, and waist circumference using two sets of criteria. The normal-weight group consisted of the participants with BMI between 20 and 25 and who met at least one of the following two criteria: fat percentage below 33 and waist circumference below 80 cm [17]. The obese group consisted of the participants with BMI 30 or over (WHO BMI guidelines) and who met at least one of the following two criteria: body fat percentage 33 or over [17] and waist circumference 80 cm or over. As two of the 98 participants from the FINALE-Health study had a BMI under 20 and 62 had a BMI between 25.1 and 29.9, these 64 participants were not included in the subsample within this nested study. All subjects gave their written informed consent when accepting participation in the study. The project was ethically approved by the Central Denmark Region Committees on Biomedical Research Ethics (M-20090050) and qualified for registration in the International Standard Randomized Controlled Trial Number Registry (NCT01015716).

### 2.2. Objective Measures

BMI, body fat percentage, and waist circumference were used to estimate body composition [18]. BMI was calculated as body weight divided by the squared height.* Body weight* was measured while wearing light clothes, but without socks and shoes. One kilogram was subtracted from the weight measure to compensate for clothing.* Height* was measured to the nearest mm without shoes.* Body Fat* percentage was measured using a bioimpedance device (TANITA SC-330), which was set to “standard” while body frame and the participant's age, height, and gender were entered.* Waist circumference* was measured over the umbilicus standing up and with clothes on, using an ergonomic circumference measuring tape (Seco 203 Girth measuring tape) and clothes thickness was noted.

### 2.3. Diet Recordings

The dietary fiber intake was estimated based on dietary records. All participants were given a dietary record with schemes to record type and quantity of food intake during nine days and instructed not to change their eating habits during the nine-day recording period. The participants had nine schemes, one for each day. Each scheme had five columns with headings saying (a) time, (b) food/fluid intake, (c) number of, (d) weight/quantity of, and (e) remarks. Participants were informed to accurately record the total intake of food and beverages and received detailed verbal and written instructions and information on how to register with the greatest possible precision. They were also handed out an example of a completed diary, so the participants had a clearer view of how the dietary records should be filled out. The program “Master Dietician System” was used to calculate the intake of macro- and micronutrients. The “Master Dietician System” is used all over Denmark by dietitians working in public hospitals and is said to be very precise by dietitians [19]. The “Master Dietician System” is limited to energy teams and distribution; hence, it does not report food types. Based on type and weight of each consumed piece of food, fed into the program, the participant's average energy intake over each 24 hours was calculated, and the distribution of all the nutrients: fatty acids, carbohydrates, and proteins enables calculation of the intake of specific nutrients. The mean grams (g) of dietary fiber intake were calculated in relation to the total megajoules (MJ) and expressed as average daily intake in g/MJ per day.

### 2.4. Estimation of Underreporting

The critical assessment of the risk of underreporting of total energy consumption is based on the model introduced by Goldberg et al. [20]. Goldberg's model is dependent on a comparison of reported energy intake with the estimated energy expenditure. Energy expenditure is for each individual calculated as the basal metabolic rate (BMR) and the physical activity level (PAL). BMR is defined as the energy expenditure of an individual lying at physical and mental rest in a thermoneutral environment. PAL gives an estimation of both occupational and leisure activity. BMR constitutes about 45 to 70 percent of the Total Energy Expenditure (TEE) in adults, and PAL constitutes about 20 to 40 percent of TEE [21]. The diet-induced thermogenesis is not included in the calculations, as the influence on TEE is much smaller. The equations proposed by Schofield in 1985 [15], backed up at WHO in 1985, and retained by WHO in 2001 [21] are used. The equation uses age, gender, and body weight of the participants. As present study is performed on females, it gives the fact that females aged 19–30 have a BMR: MJ/day (0.062 w + 2.036) and females aged 31–60 have a BMR: MJ/day (0.034 w + 3.538) [15]. PAL is set to 1.8, indicating a day including both standing and moving around [22]. The estimation of underreporting is obtained by calculating the ratio between the reported energy intake (EI) and the participants' estimated BMR (EI/BMR).

### 2.5. Statistical Analysis

Parametric unpaired *t*-test with equal variance is used to analyze differences between the two groups. The population is defined by a group of 34 health care workers from the FINALE-Health study fulfilling either the criteria for normal-weight or obesity presented in Table 1. A post hoc power analysis with 80% significance level showed that a group difference of 0.60 g in dietary fiber intake could be detected with a significance level of alpha < 0.05. This corresponds to 20% of recommended daily intake of fibers and is regarded as of clinical significance.

## 3. Results

### 3.1. Description

The description of the two groups is presented in Table 1, showing no significant difference in age and height between the groups.

### 3.2. Dietary Fiber Intake

Table 2 presents the average intake in kJ/day and percentage of proteins, carbohydrates, fat, and dietary fiber. The obese group generally had a diet that was slightly higher in fat and significantly lower in dietary fiber (*p* = 0.012), when compared to the normal-weight group (Table 2). Only the difference in dietary fiber intake was significant between the two groups.

**Figure 1:**
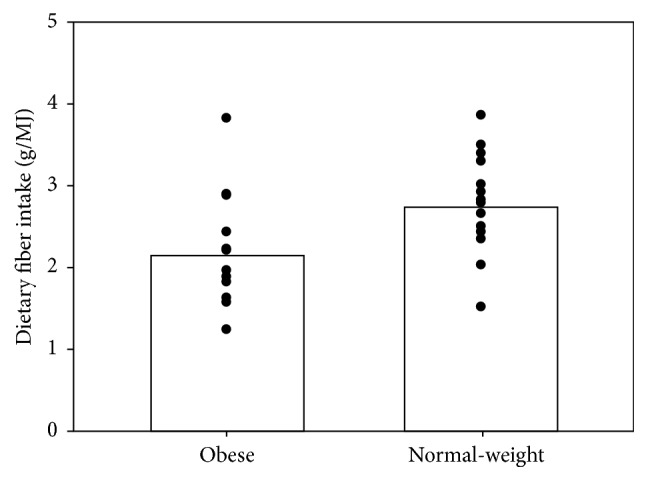
Distribution of individual dietary fiber intake (g/MJ) in the obese and normal-weight subjects and average value.

The intake of dietary fiber ranged from 1.51 to 3.86 g/MJ in the normal-weight group and from 1.23 to 3.83 g/MJ in the obese group; for individual values see Figure 1. *p* value of the fiber intake in the two groups is *p* = 0.012. The daily-recommended dietary fiber intake is 3 g/MJ or 25–35 g/day.

### 3.3. Underreporting

The average energy intake relative to the basal energy intake, calculated as the EI/BMR value, is in the normal-weight group 1.37 and for the obese group 1.35 (Table 3). The difference in intake between the two groups is not statistically significant.

**Figure 2:**
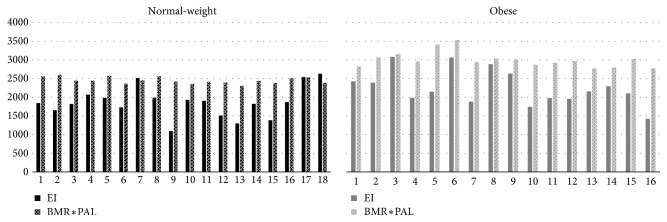
Reported energy intake (EI) and basal metabolic rate adjusted according to physical activity level (BMR*∗*PAL) for the normal-weight and obese individuals included in the study.

The low energy intake per basal metabolic rate quota EI/BMR in both groups is indicative of a systematic underreporting of energy intake in both groups (Figure 2).

## 4. Discussion

In this study, we present data on low-income low-education female workers divided into two groups: obese and normal-weight elucidating their dietary habits. As we wanted to seek for differences between the two groups, we excluded the overweight group. The present results show a significant difference in dietary fiber intake between the obese and the normal-weight female health care workers. In addition, the dietary fiber intake is low for both groups, when compared to recommended daily fiber intake [8]. The recorded dietary intake reveals that the participants probably have been underreporting as the reported food intake is less than the limit of the recommended reference value of 1.8 EI/BMR [20, 23]. The low basal metabolic rate quota EI/BMR in both groups is indicative of a systematic underreporting in both groups and shows that normal-weight individuals are as prone to underreporting as obese individuals, contradictory to findings in other studies [14].

The present result of obese individuals having a significantly lower dietary fiber intake compared to normal-weight is in coherence with previous studies concerning more weight and socioeconomically mixed populations. Based on a three-day diet diary Alfieri et al. [24] from a population of 150 individuals found a total fiber intake of 2.33 g/MJ, 1.74 g/MJ, and 1.6 g/MJ in normal-weight (BMI 20–25), moderately overweight (BMI 25–30), and extremely overweight subjects (BMI > 35), respectively [24]. High dietary fiber intake was in addition positively associated with high education [24]. Howarth and colleagues [25] conducted a study on two-day diet records derived from a data base of a mixed population of 4539 young and middle aged men and women [25] also showing a correlation between diet composition and BMI. The low dietary fiber intake was set at less than 1.5 g/MJ and was associated with overweight [25]. The dietary fiber intake in the present study was somewhat higher than the intake in individuals with high BMI in the previous studies. There may be several reasons for the discrepancy but plausible reason is dietary differences between the countries, where the studies were conducted, although the exact discrepancies in dietary habits between countries need to be further elucidated.

According to the Nordic nutrition recommendations, 2012, a recommended intake of dietary fiber is 3 g/MJ [8]. An adequate intake of dietary fiber reduces the risk of constipation and contributes to a reduced risk of colorectal cancer and several other chronic diseases such as cardiovascular disease and type 2 diabetes [6]. Moreover, fiber-rich foods help in maintaining a healthy body weight. The 0.58 g/MJ difference in fiber intake between the normal-weight and the obese groups shows the relation between obesity and dietary choice, that is, intake of dietary fiber. The clinical relevance of the 0.58 g/MJ difference in fiber intake is difficult to specify, but there is evidence of an association between increased fiber intake and health status, no matter if the fiber intake is over or under 3 g/MJ [6] per day. An example is a study from Harvard University where the researchers found an association between increased fiber intake and decreased prevalence of breast cancer [7].

Daily energy intake and dietary fiber intake per energy unit in the present study are based on a nine-day diet diary. Dietary records are considered to be a good method for assessing diet intake [26] but several studies have found misreporting when individuals have to write down their daily intake of food [27, 28]. Underreporting has been shown to be higher in groups with low socioeconomic status compared to high socioeconomic status and is normally biased towards underreporting of energy intake [12, 13]. A misreporting of the daily energy intake can lead to uncertainties in the calculated values for a specific nutrient, and it can have an impact on the results of fiber intake obtained in the current study.

On the other hand, underreporting is in many cases specific, since amounts of fat and sugar rich foods suffer more from underreporting than fruit and vegetables [27, 28]. In addition, previous studies specifically identify underreporting between meals snacks [27, 29]. As health care workers typically have many small breaks in their daily work this may tempt them to consume such quick snacks that tend to be forgotten in the reporting. This is somehow supported in the present study, indicating a general systematic underreporting of the actual energy intake in both groups, but the present data do not allow a determination of which type of food is underreported and therefore it is also unknown how it may bias the estimation of fiber intake. The tendency to underreport is only slightly larger in the obese group while previous studies generally report greater underreporting in obese than in normal-weight individuals [14] or an overall underreporting independent of BMI [30].

The underreporting may also be due to the measurement in itself imposing behavioral changes. When conducting diet recordings, the participants weight their food and reflect on the food-mass before consumption. While this may lead to less reliable results for estimation of normal daily energy intake, it also points at diet recordings as an option to nudge a healthier lifestyle. This could enable an increased intake of dietary fiber shown to have positive effects on public health diseases such as type 2 diabetes [31, 32], heart diseases, and cancer [6, 33]. Dietary habits together with moderate exercise have shown having an effect on cardiovascular health, extending life span, and reducing the risk of cancer [34]. Numerous studies state that the “healthy eating and sport” combination is essential for a state of good health because it can trigger metabolic responses involved in body regulation. See, for instance, Francavilla et al. (2007) [35].

Health beneficial counseling has shown positive effects in populations of overweight women [36]. Counseling educates the participants and helps them become more aware of the health issues connected to their food intake. Participants also learn healthier eating habits resulting in weight reduction [36]. In future studies it may be of interest to elucidate the individual awareness regarding dietary intake in order to understand all the contributing factors for overweight and obesity within a population.

Health care workers conduct physically hard work and have a high prevalence of work related pain disorders [37], cardiovascular diseases [38], long-term sick leave [39], and early retirement [40].

Being obese apart from being a risk factor for cardiovascular disease causes an additional strain to muscle and joints and may contribute to pain conditions. Introducing weight loss programs at the workplaces may therefore contribute to a reduction in pain. The advantages of health promoting interventions at the workplace have been presented in a recent study, where introduction of dietary advice and physical exercise among health care workers have resulted in increased productivity and less sick leave [41]. The present study is a cross-sectional study but the result on a low fiber intake may encourage targeted studies introducing lifestyle interventions including dietary advice on fiber intake as a health promoting activity at workplaces.

The strength of this study is that the study is conducted with a solid diet registration of nine days on a well-defined subpopulation having the same everyday work exposure. Hence, differences in non-leisure time activity are insignificant. Weaknesses are the relatively small population and the cross-sectional design limiting interpretation of the cause-effect relation.

## 5. Conclusion

The current study shows a positive association between low dietary fiber intake and a high BMI, in a population of low educated women working within health care. In general, food intake was underreported and compared to the recommended daily fiber intake; the reported dietary fiber intake was low in both the normal-weight and obese group. As a high proportion of health care workers are obese, the results indicate a clear potential benefit from including dietary advice especially on fiber intake in order to increase general health and decrease weight within this group.

## Figures and Tables

**Table 1:** Description of the normal-weight and the obese group.

	Normal (*n* = 18)	Obese (*n* = 16)	*p* value^*∗*^
Age, years	47.6 ± 8.8	44.7 ± 8.3	0.34
Height, cm	166.9 ± 5.2	166.3 ± 6.1	0.74
Weight, kg	62.8 ± 5.8	98.0 ± 11.0	<0.01
BMI, kg × m^2^	22.5 ± 1.3	36.5 ± 4.3	<0.01
Fat percentage, %	28.8 ± 3.2	42.3 ± 3.5	<0.01
Waist circumference, cm	78.9 ± 7.7	108.8 ± 11.9	<0.01
Fat mass, kg	18.1 ± 2.9	41.6 ± 7.4	<0.01
Fat free mass, kg	44.6 ± 4.1	56.3 ± 4.9	<0.01

^*∗*^Significant  *p* value, *p* > 0.05, in differences between the normal-weight and the obese group.

**Table 2:** Intake in kJ/day and percentage of proteins, carbohydrates, fat, and dietary fiber.

	Intake kJ/day	Proteins%	Fat%	Carbohydrates%	Dietary fiber g/MJ
Normal- weight	7832.0 ± 1728.4	14.7 ± 2.5	30.9 ± 5.8	50.8 ± 7.7	2.73 ± 0.65
Obese	9484.4 ± 1966.8	14.4 ± 2.1	33.5 ± 4.2	49.3 ± 5.8	2.15 ± 0.64

**Table 3:** Total calories from nine-day diary. Reported daily intake (EI) in kcal, basal metabolic rate (BMR), and energy intake/basal metabolic rate (EI/BMR) calculated from reported calorie intake according to Schofield et al. [15]. Total energy expenditure (TEE) (kcal) according to physical activity level (PAL) of 1.8 for each group. All values are presented as average of each group with standard deviation in brackets.

	EI (kcal)	BMR (kcal)	TEE (kcal)	EI/BMR
Normal-weight	1865 (±400)	1361 (±49)	2450 (±83)	1.37
Obese	2258 (±453)	1667 (±115)	3001 (±207)	1.35
